# T-Cell Density at the Invasive Margin and Immune Phenotypes Predict Outcome in Vulvar Squamous Cell Cancer

**DOI:** 10.3390/cancers14174246

**Published:** 2022-08-31

**Authors:** Eike Burandt, Niclas C. Blessin, Ann-Christin Rolschewski, Florian Lutz, Tim Mandelkow, Cheng Yang, Elena Bady, Viktor Reiswich, Ronald Simon, Guido Sauter, Sven Mahner, Nikolaus de Gregorio, Rüdiger Klapdor, Matthias Kalder, Elena I. Braicu, Sophie Fürst, Maximilian Klar, Hans-Georg Strauß, Katharina Prieske, Linn Wölber

**Affiliations:** 1 Institute of Pathology, University Medical Center Hamburg-Eppendorf, 20246 Hamburg, Germany; 2 Department of Obstetrics and Gynecology, University Hospital, LMU Munich, 81377 Munich, Germany; 3 Department of Gynecology, University Hospital Ulm, 89070 Ulm, Germany; 4 Klinikum am Gesundbrunnen, SLK-Kliniken Heilbronn GmbH, 74078 Heilbronn, Germany; 5 Department of Gynecology and Obesterics, Hannover Medical School, 30625 Hannover, Germany; 6 Department of Gynecology, Medical Center Philipps University Marburg, 35043 Marburg, Germany; 7 European Competence Center Ovarian Cancer, Charité, Campus Virchow Klinikum, Charité-University Hospital, 13353 Berlin, Germany; 8 Department of Gynecology, University of Freiburg, 79106 Freiburg im Breisgau, Germany; 9 Department Gynecology and Policlinic, University Hospital Martin-Luther-Universität Halle-Wittenberg, 06120 Halle, Germany; 10 Department of Gynecology, University Medical Center Hamburg-Eppendorf, 20246 Hamburg, Germany; 11 Colposcopy Clinic at the Jerusalem Hospital Hamburg, 20357 Hamburg, Germany; 12 Mildred Scheel Cancer Career Center HaTriCS4, University Medical Center Hamburg—Eppendorf, 20246 Hamburg, Germany

**Keywords:** vulvar squamous cell cancer, immunohistochemistry, TILs

## Abstract

**Simple Summary:**

Quantification of tumor infiltrating lymphocytes (TILs) in the cancer microenvironment has become of increasing interest in immuno-oncology and has been therefore studied extensively in all “high prevalence” cancer entities. However, only little is known about TILs infiltration in rare cancer entities, such as vulvar cancer. Here, we have studied an exceptional large multicenter cohort of 530 vulvar squamous cell cancer specimens using immunohistochemistry. The study was conducted using large tissue section to quantitate the CD3^+^/CD8^+^ T-cell density along the invasive margin compared to the center of the tumor. The CD3^+^ T-cell density at the invasive margin showed the strongest prognostic value. Moreover, analysing the interplay between both T-cell densities—at different locations—enabled us to identify three major immune phenotypes that showed strong prognostic relevance. Accordingly, the sole analysis of CD3^+^ T-cells at the invasive margin—rather than a cumbersome immune score—predicts patient’s outcome in vulvar cancer.

**Abstract:**

Background: Although quantification of tumor infiltrating lymphocytes (TILs) has become of increasing interest in immuno-oncology, only little is known about TILs infiltration in the tumor microenvironment and its predictive value in vulvar cancer. Methods: Immunohistochemistry and automated digital image analysis was applied to measure the densities of CD3^+^ (DAKO, #IR503) and CD8^+^ (DAKO, #IR623) TILs at the invasive margin and in the center of 530 vulvar squamous cell cancers. Results: An elevated density of CD3^+^ T-cell at the invasive margin was significantly associated with low tumor stage (*p* = 0.0012) and prolonged survival (overall survival [OS] *p* = 0.0027, progression free survival [PFS] *p* = 0.024) and was independent from tumor stage, nodal stage, grade, and HPV-status in multivariate analysis (*p* < 0.05). The prognostic impact of CD3^+^ cells in the center of the tumor was weaker compared to the invasive margin (OS *p* = 0.046, PFS *p* = 0.031) and lacking for CD8^+^ T-cell densities at any location (*p* ≥ 0.14 each). Unsupervised clustering of CD3^+^ and CD8^+^ T-cell densities identified three major subgroups corresponding to the immune desert (137 patients), immune excluded (220 patients) and immune inflamed phenotypes (133 patients). Survival analysis revealed a particular poor prognosis for the immune desert phenotype for OS (*p* = 0.0071) and PFS (*p* = 0.0027). Conclusion: Our data demonstrate a high prognostic value of CD3^+^ T-cells at the invasive margin and immune phenotypes in vulvar squamous cell cancer.

## 1. Introduction

Vulvar cancer represents a rare gynaecological malignancy with steadily increasing incidence [1,2,3]. Despite therapy, about 50% of nodal positive patients recur within five years after diagnosis [4]. Treatment of advanced stages or recurrent disease usually consists of multimodal approaches with combinations including surgery, radiotherapy, and/or chemotherapy, although there is no widely accepted standard treatment for recurrent or metastatic disease [2,5].

In the era of immune checkpoint inhibitor therapy, the number of therapies and indications that gain approval by the US Food and Drug Administration is steadily increasing [6]. Immune checkpoint therapy has been already approved for more than 10 different solid tumor entities, such as breast cancer, malignant melanoma, NSCLC, renal cell, gastric, urothelial, and head and neck cancer [7,8,9,10,11]. Furthermore, rare cancer entities such as vulva cancer also represent a promising candidate for drugs targeting immune checkpoint molecules. Thus, several phase I and phase II clinical trials using immune checkpoint inhibitors—mostly within basket designs—in vulvar cancer are currently ongoing, such as NCT02054806, NCT02488759, NCT03452332, NCT03277482, NCT02379520, NCT02054806, NCT03439085, NCT02834013, NCT02628067, NCT04430699, and NCT04357873. Some clinical trials have already reported partial response to PD-L1 inhibitors [12] (NCT02054806) and PD-1 inhibitors [13] (NCT02488759). Although there are several ongoing clinical trials investigating immunotherapy in vulvar cancer, only little is known about the distribution of tumor infiltrating lymphocytes (TILs) in the tumor microenvironment of vulvar cancer and their potential predictive value. The fact that studies on 65 to 286 vulvar squamous cell cancer revealed a favorable prognosis for patients showing a high degree of TILs infiltration [14,15,16,17,18] underlines that an anti-tumor immunity to control tumor growth can also be present in vulvar cancer. The understanding of TILs in the tumor microenvironment might not only extend the limited amount of established prognostic parameters [5,19] in vulvar cancer, it could also improve the response to immune checkpoint therapy [20,21,22,23].

To study the spatial composition and distribution of TILs, relative to the cancer cells, in vulvar carcinomas, the infiltration of CD3^+^ and CD8^+^ T-cell was analyzed at the invasive margin and in the center of the tumor. The large sections analyzed in this study consisted of 530 vulvar carcinomas—not treated with immune checkpoint inhibitors—from an exceptionally big and clinically well characterized multicenter cohort [4].

## 2. Materials and Methods

### 2.1. Patients and Tissues

Formalin-fixed paraffin embedded tumor tissue samples from 637 patients with vulvar squamous cell cancer from a sub-study of the AGO (Arbeitsgemeinschaft Gynäkologische Onkologie) -CaRE (Chemo and Radiotherapy in Epithelial Vulvar Cancer) -1 study were included in this study. The AGO-CaRE-1 study is a large retrospective study, focusing on the evaluation of treatment pattern and prognostic factors in vulvar cancer. All patients with the diagnosis of invasive vulvar cancer stage >pT1a independent of the mode and initial place of treatment could be included by the participating institutions. 1618 patients with stage IB-IV VSCC [Union for International Cancer Control (UICC) version 6], treated between 1998 and 2008 at 29 AGO cancer centers in Germany, were included. Data on the HPV vaccination status are not available, but the effect should not affect this study because 74% where operated before 2006. Patient data collection was performed retrospectively between February and December 2011. Tumor characteristics as well as aspects of surgical and nonsurgical treatment were collected. Documentation and analysis were done through a specifically designed centralized database by the AGO-study-group. The AGO-CaRE-1 study, and the translational sub-study, were approved by each local Ethics Committee (leading vote: Hamburg (reference number PV3658)) and registered with ClinicalTrials.gov (NCT01304667). Detailed information about the recruitment and data collection were published by Mahner et al. [4] (For patient characteristics see Table S1).

### 2.2. Immunohistochemistry

Freshly cut 4 µm tissue sections were stained for CD3^+^ T lymphocytes and the subset of CD8^+^ cytotoxic T-cells. Following deparaffinzation, slides were exposed to heat-induced antigen retrieval for at 98 °C in pH 9 target retrieval solution (Agilent, Santa Clara, CA, USA) in a PT Link pre-treatment module (Agilent) and stained in an Autostainer Link 48 device (Agilent) using primary antibodies against CD3 (Dako, rabbit polyclonal antibody, Santa Clara, CA, USA; #IR503; undiluted) and CD8 (DAKO, mouse monoclonal antibody, #IR623, undiluted). Protocol steps were performed according to the manufacturer′s directions and include 5 min peroxidase blocking (Agilent REAL) and 20 min of primary antibody incubation at room temperature followed by visualization of the bound antibody using the EnVision Flex Kit (Agilent). 

### 2.3. Definition of Analyzed Compartments and Quantification of CD3 and CD8 Immunostaining

Slides were digitalized using Leica′s Aperio VERSA 8 automated microscope. A trained pathologist annotated the invasive margin and the center of the tumor in each digital image. The invasive margin was defined as the area expanding 300 µm into the stroma and 50 µm into the tumor measured from the stroma-tumor-borderline. The center of the tumor was defined as a tumor area remote from the stroma-tumor-borderline [24]. Slides with obvious staining artefacts or damaged tissue or lack of unequivocal invasive margin/tumor center regions were excluded from further analysis. The remaining slides matched the inclusion criteria for further image analysis of the invasive margin and center of the tumor. The “Image Scope” software package (Leica Microsystems Wetzlar, Germany) was used to train a machine learning algorithm for cell segmentation of bright field immunohistochemistry. The number of stained cells and the area of the annotated region was measured in each annotation of IM and CT and used for the calculation of the density of stained cells (number of cells per square mm). The whole procedure is visualized in Figure S1. The difference between the CD3^+^ T-cell density and CD8^+^ T-cell density was calculated to estimate the density of CD4^+^ T-cells.

### 2.4. Definition of Immune Phenotypes and Cluster Analysis

An unsupervised cluster analysis and a slide review by a pathologist were utilized to distinguish the previously described immune phenotypes: “immune inflamed”, “immune excluded” and “immune desert” phenotype [20,23,25]: The immune inflamed phenotype is characterized by a high density of TILs at the invasive margin as well as in the center of the tumor and the immune desert phenotype is characterized by a low density of TILs in both compartments [23,26]. A unique feature of the immune excluded phenotype is a high density of TILs at the invasive margin, while there is a paucity of TILs found in the center of the tumor [23,26]. A pathologist categorized all suitable cases in immune “inflamed”, “desert”, “excluded” and “unidentified” based on the HE stains and the available immunohistochemical stains in a consecutive slide review according to Ribbbat-Idel et al. [27]. The “Immune inflamed” phenotype was found in case of diffusely distributed tumor infiltrating lymphocytes in more than 5% of all tumor compartments. The “Immune excluded” phenotype was identified if less than 5% of the center of the tumor consisted of diffusely distributed tumor infiltrating lymphocytes but more than 5% of the invasive margin consists of lymphocytes. The “Immune desert” phenotype was characterized by less than 5% lymphocytic infiltration in all tumor compartments. Unsupervised clustering of CD3^+^ and CD8^+^ densities (cells/mm^2^) at the invasive margin and in the center of the tumor, using the “hclust package” in R (The R foundation) [28], was also applied to identify the three major immune phenotypes. 

### 2.5. Statistics

R version 3.6.1 (The R foundation) [28] and JMP Pro 14 software package (SAS Institute Inc., Cary, NC, USA) [29] were used in this study. To study the relationship between cell densities and clinico-histopathological parameters, contingency table analysis and Chi-square test (likelihood) were used. Analysis on overall survival (OS) and progression-free survival (PFS) were performed using Kaplan–Meier Estimates (R “survival” [30] package) and the log-rank test was applied to assess differences between the three groups, that were allocated according to the 80%- as well as 20%-quantile, in Kaplan–Meier Estimates. To estimate whether the prognostic impact of the CD3^+^ and CD8^+^ T-cell density was independent from tumor stage, nodal stage, histological grade and HPV-status, multivariable Cox proportional hazard models were calculated.

## 3. Results

### 3.1. T-Cell Densities

**Figure 1:**
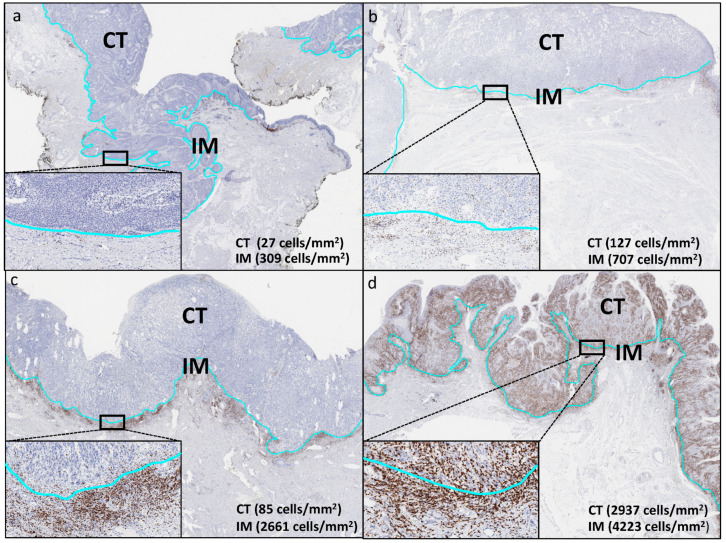
T-cell infiltration patterns in vulvar cancer. Representative images of an immune desert (**a**); immune excluded (**b**,**c**), and an immune inflamed phenotype (**d**) are depicted. The light-blue line indicates the invasive margin (IM) that represents the outer edge of the center of the tumor (CT). The images are taken at 5× magnification.

530 of 637 (83%) stained slides matched the inclusion criteria of this study and contained lymphocytes positive for CD3 and CD8 in the invasive margin (IM) as well as in the center of the tumor (CT). The remaining 107 cases were excluded due to missing tissue, absence of unequivocal cancer cells or the lack of an IM/CT. The T-cell density at the IM (CD3: 0.2–5424 cells/mm^2^, CD8: 2–3385 cells/mm^2^) and in the CT (CD3: 0.5–4650 cells/mm^2^, CD8: 1–3115 cells/mm^2^) was highly variable between patients. Examples of individual cases are given in Figure 1a–d. On average, there were significantly more CD3^+^ and CD8^+^ T-cells located at the invasive margin (CD3: 1772 ± 1105, CD8: 769 ± 644 cells/mm^2^) than in the center of the tumor (CD3: 518 ± 570, CD8: 301 ± 445 cells/mm^2^, *p* ≤ 0.0001). This T-cell predominance at the invasive margin was seen in 96% (CD3) and 91% (CD8) of individual cancers.

### 3.2. Association with Tumor Phenotype and Patient Survival

**Figure 2:**
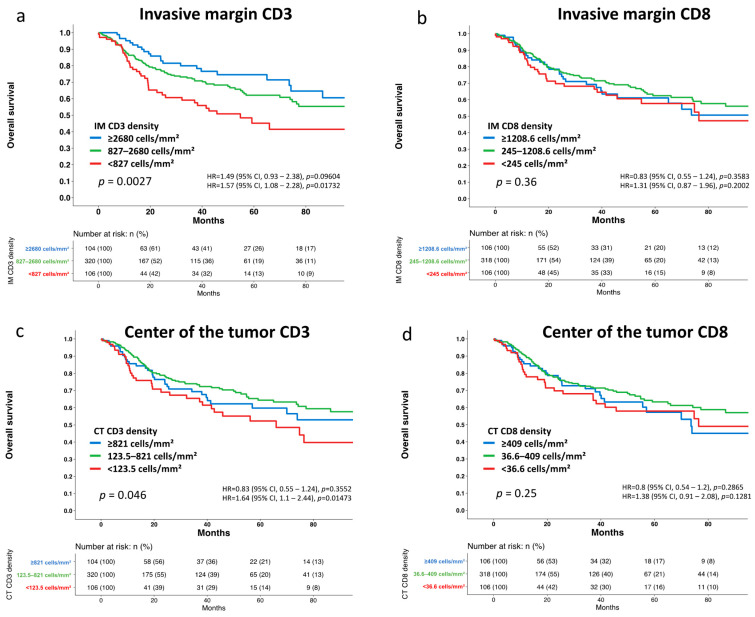
Kaplan–Meier Estimates for overall survival in vulvar cancer. Prognostic impact of the CD3^+^ and CD8^+^ T-cell density at the invasive margin (**a**,**b**) as well as in the center of the tumor (**c**,**d**).

Follow-up data on overall survival (OS) and progression free survival (PPS) were available from all 530 analyzed patients (Table S1). An elevated CD3^+^ T-cell density at the IM was significantly associated with low tumor stage (*p* = 0.0001, Table 1) and prolonged survival (overall survival (OS) *p* = 0.0027, progression free survival (PFS) *p* = 0.024, Figure 2, Figure S2). The 2-years OS and PFS rate was significantly different between the group with a high (OS: 82%, PFS: 65%), a moderate (OS: 76%, PFS: 55%), and a low CD3^+^ T-cell density at the IM (OS: 64%, *p* = 0.008, PFS: 44%, *p* = 0.02). A high CD3^+^ cell density at the center of the tumor (CT) was not significantly related to tumor stage but linked to prolonged survival, although this association was somewhat weaker (OS *p* = 0.046, PFS *p* = 0.031) than seen for the tumor periphery (Table 1, Figure 2, Figure S2). No associations were found between clinicopathological parameters and the CD8^+^ density in the center of tumor (*p* > 0.16 each, Table 1, Figure 2, Figure S2). The estimated CD4^+^ T-cell density at the IM was significantly associated with low tumor stage (*p* < 0.0001), negative nodal stage (*p* = 0.024) and linked to prolonged overall and progression free survival (≤0.012, Table S2, Table S3, Figure S4). The CD3^+^ T-cell density at the invasive margin (OS: *p* = 0.017, PFS: *p* = 0.044) and the center of the tumor (OS: *p* = 0.004, PFS: *p* = 0.006) was an independent prognostic factor (Table 2).

### 3.3. Cluster Analysis of the T-Cell Density

**Figure 3:**
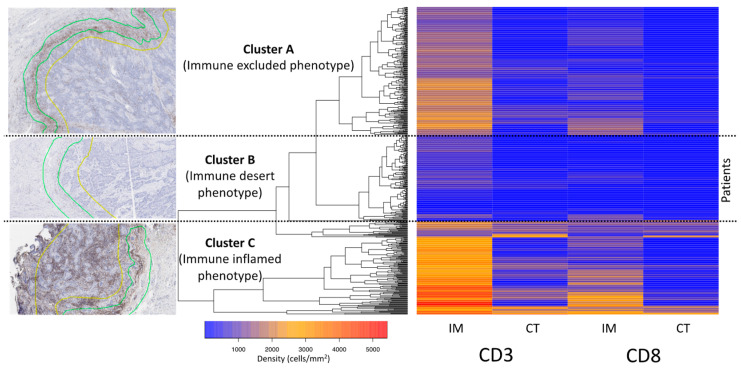
Unsupervised hierarchical cluster analysis of vulvar cancer samples. The two-way (T-cell densities against samples) unsupervised hierarchical cluster of 530 vulvar cancer samples and the CD3^+^/CD8^+^ T-cell densities (rows) at the invasive margin (IM) and in the center of the tumor (CT) is shown. Representative images of the three major clusters are depicted next to the corresponding clusters.

**Figure 4:**
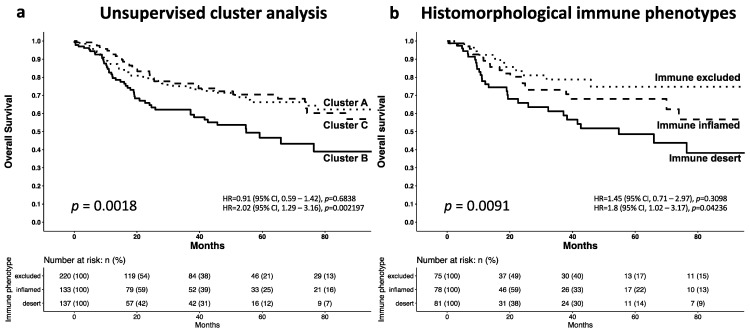
Kaplan–Meier Estimates for overall survival among patients with different immune phenotypes. Prognostic impact of the clustered (**a**) and histomorphological identified; and (**b**) immune phenotypes.

To search for patterns in the measured T-cell densities, an unsupervised clustering approach was applied. Three major clusters (A: *n* = 222, B: *n* = 137, C: *n* = 133, Figure 3) were identified. In cluster A, 75 (34%) patients had an inflamed IM (CD3: >1500 cells/mm^2^, CD8: ≥50 cells/mm^2^) but lacked immune infiltration in the CT (CD3: <300 cells/mm^2^, CD8: <200 cells/mm^2^). In cluster B, 81 (59%) patients showed neither immune infiltration in the IM (CD3: <900 cells/mm^2^) or in the CT (CD3: <150 cells/mm^2^). In cluster C, 78 (59%) patients had strong inflammation in the IM (CD3: >2300 cells/mm^2^) and the CT (CD3: >650 cells/mm^2^). Accordingly, cluster A met the criteria of an immune excluded phenotype, Cluster B corresponded to the criteria of an immune desert phenotype and cluster C showed an immune inflamed phenotype. Survival analysis of the clustered cases revealed a particular poor prognosis for the immune desert cluster B (OS *p* = 0.0018, Figure 4a; PFS *p* = 0.012, Figure S3a). A histopathological review of all cases had identified 75 immune excluded, 81 desert, and 78 inflamed phenotypes. Survival analysis of these three immune phenotypes also revealed a poor prognosis for the immune desert phenotype (overall survival *p* = 0.0091, Figure 4b; progression free survival *p* = 0.0043, Figure S3b). 

## 4. Discussion

Successful analysis of CD3^+^ and CD8^+^ tumor infiltrating lymphocytes (TILs) in 530 vulvar squamous cell cancers (squamous cell carcinomas of the vulva) identified that the CD3^+^ TIL density, but not the CD8^+^ TIL density, was independently associated with patient’s outcome. This appears counterintuitive at first sight, since CD8^+^ lymphocytes are known to be the most relevant immune cell population responsible for the killing of tumor cells [31,32]. However, previous studies analyzing cohorts of 65, 76, 85 and 286 vulvar squamous cell cancers also failed to find statistical associations between the number of CD8^+^ cytotoxic T-cells and patient outcome [14,15,16,17]. The only study suggesting a link between high number of CD8^+^ T-cells and favorable prognosis had involved only 21 vulvar cancer patients [33]. Hence, these results suggest that other T-lymphocyte subpopulations than CD8^+^ T-cells may have a significant prognostic relevance in vulvar cancer. For example, the density of helper T-cells have been linked to patient prognosis in vulvar cancer [14,15]. Helper T-cells were also often found to have prognostic impact in several other cancer entities such as breast [34,35], colorectal [36], pancreatic [37] and non-small cell lung cancer [38]. In agreement with these studies our estimated CD4^+^ T-cell density at the invasive margin was associated with improved patient’s outcome although this parameter may lack precision because it was calculated by subtraction of CD8^+^ T-cells from CD3^+^ T-cells quantified on a consecutive TMA section. The lack of an association between the density of CD8^+^ T-cells and patient prognosis does not imply that CD8^+^ T-cells are not relevant in vulvar cancer. It is possible that specific subpopulations such as active, exhausted or expanding CD8^+^ T-cells are more strongly linked to patient outcome than the entirety of these cells. This corresponds to studies showing that an elevated density of CD8^+^Tim3^+^ cells [39,40] and Granzym B^+^ cytotoxic T-cells [18] were recently shown to be linked to prolonged survival in vulvar cancer. 

Our results also showed that the T-cells at the invasive margin appeared to be more prognostically relevant than T-cells in the tumor periphery. The fact that the CD3^+^ T-lymphocyte density at the invasive margin was identified as the major parameter for patient′s outcome in this study is consistent with data from several studies in other tumor types. For example, Galon et al. found a significant impact of CD3^+^ TILs at the invasive margin on patient′s survival in all three analyzed cohorts of colorectal cancer patients, but only one of three cohorts showed a prognostic significance for CD3^+^ TIL density in the center of the tumor [41]. In agreement with our results a recent study has shown prognostic relevance for CD3^+^ cells at the invasive margin, but not for CD3^+^ T-cells in the center of the tumor, in 283 microsatellite stable colorectal cancers [42]. The pivotal role of TILs at the invasive margin [43] might be explained by these cells representing an “immune wall” which protects the host from vascular and lymphatic channel invasion by tumor buds [44]. In contrast to several TILs scores in other tumor entities such as the Immunoscore in colorectal cancer, which includes four TIL variables (i.e., CD3^+^IM, CD3^+^CT, CD8^+^IM, CD8^+^CT) [41], the data from this study demonstrated that CD3^+^ alone at the invasive margin provides robust prognostic information. 

An unsupervised cluster analysis based on lymphocyte densities in different tissue compartments had identified immune cell patterns that correspond to the “immune desert”, “immune excluded” and “immune inflamed” phenotypes which were initially described by Chen and Mellman [23]. The fact that these patterns are clinically relevant in vulvar squamous cell cancer is supported by the strong prognostic impact of these patterns irrespective of whether these were identified by cluster analysis or by “pattern recognition” of a pathologist. Both approaches revealed a particular poor prognosis for the immune desert phenotype and no significant difference between the immune excluded and immune inflamed phenotype in vulvar cancer. The poor prognosis of tumors showing an immune desert phenotype might be due to insufficient T-cell priming [45,46] and immunological ignorance [45,46], which results in the absence of a pre-existing anti-tumor immunity, particularly the lack of tumor specific effector T-cells [20,23,45,47].The fact that the prognosis of the inflamed and excluded immune phenotype appeared similar, and superior to the immune desert phenotype, suggest equally effective immune evasion mechanisms, which such cancers must inevitably have developed—in order to exist—to overcome the pre-existing anti-tumor immunity. Accordingly, mechanical barriers [48] (e.g., stromal fibrosis [49], vascular formation [50]) and functional barriers [48] (e.g., metabolic barriers [51], TGF-ß [52], CD47-SIRPalpha signaling axis [53], immune checkpoint expression [23]) along the invasive margin were found preventing TILs from entering the tumor center in the immune excluded phenotype. In the inflamed phenotype, the immune evasion is mainly driven by immunosuppressive cell-cell interaction between PD-L1 expressing cells [54], regulatory T-cells [55], cancer associated fibroblasts [23,56] and anti-tumor TILs to render ineffective the pre-existing anti-tumor immune response. This might suggest that even though the immune evasion strategies are located spatially separate in inflamed and excluded phenotypes the effectiveness remains similar as seen by the same prognostic relevance of both immune phenotypes in squamous cell carcinomas of vulvar and tongue [57]. 

It is of note that our patients represent a historic tumor cohort not treated by immune checkpoint inhibitors. However, the prognostic impact demonstrated for CD3^+^ TILs and immune phenotypes described in this study suggests that the specific immune response to individual vulvar carcinomas has a clinically relevant impact on the course of the disease. It is thus very likely, that drugs with an impact on the immune cell capabilities to eliminate cancer cells will influence the disease course as well. Given that the probability for response to immune checkpoint therapy increases according to the degree of TILs infiltration [58] and thus a particular high response rate was seen for the immune inflamed phenotype [20,58,59], the 34% of clearly inflamed vulvar squamous cell cancers among the 530 analyzed samples might be particularly likely to respond to immune checkpoint therapy. In support of this hypothesis, response to PD-L1 inhibitors [12] (NCT02054806) and PD-1 inhibitors [13] (NCT02488759) in vulvar cancer has been reported recently. Response rate of immune checkpoint inhibitors was below 25% in two clinical trials including 5 and 18 patients with advanced vulvar cancer [12,13]. Of note, the degree of TIL infiltration and the immune phenotype was not evaluated in both studies. TILs quantification is also not part of ongoing phase 1 (NCT03452332, NCT03277482, NCT02379520, NCT02054806) and phase 2 (NCT03439085, NCT02834013, NCT02628067, NCT04430699, NCT03220009, NCT04357873) clinical trials using immune checkpoint inhibitors in vulvar cancer.

## 5. Conclusions

In conclusion, the results of the present study showed that standardized TILs quantification in vulvar cancer enables the characterization of three major immune phenotypes, which predict patient outcome according to the degree of CD3^+^ T-cell infiltration at the invasive margin. Further studies are now needed to evaluate the underlying mechanisms of the major immune phenotypes and patterns for response to immune checkpoint therapy in vulvar squamous cell cancer. The combination of multiple markers using novel multiplex fluorescence immunohistochemistry methods such as CODEX [60] or BLEACH&STAIN [61] might facilitate these future studies.

## Figures and Tables

**Table 1:** Association between the density of CD3^+^/CD8^+^ T-cells at the invasive margin/in the center of the tumor and vulvar cancer phenotype. (±Standard deviation).

Clinical Parameter	n	Density of CD3^+^ Cells (IM *) [cells/mm^2^]	*p*-Value	Density of CD3^+^ Cells (CT **) [cells/mm^2^]	*p*-Value	Density of CD8^+^ Cells (IM *) [cells/mm^2^]	*p*-Value	Density of CD8^+^ Cells (CT **) [cells/mm^2^]	*p*-Value
pT1	174	1995 (±1239)	0.0001	583 (±620)	0.0518	800 (±668)	0.0259	311 (±448)	0.1573
pT2	286	1738 (±1042)		503 (±551)		791 (±645)		312 (±461)	
pT3-4	64	1351 (±856)		388 (±445)		565 (±475)		199 (±276)	
pN-	285	1863 (±1150)	0.0638	523 (±584)	0.5712	798 (±685)	0.3459	311 (±485)	0.3150
pN+	180	1665 (±1076)		493 (±520)		741 (±571)		270 (±330)	
Grade 1	60	2068 (±1240)	0.0664	500 (±531)	0.6560	878 (±730)	0.2047	256 (±424)	0.6782
Grade 2	317	1768 (±1107)		502 (±535)		777 (±660)		298 (±417)	
Grade 3	145	1674 (±1032)		553 (±645)		707 (±534)		316 (±493)	
HPV-	244	1818 (±1104)	0.5099	520 (±586)	0.9229	819 (±667)	0.1258	296 (±399)	0.7400
HPV+	259	1753 (±1113)		525 (±572)		731 (±630)		310 (±492)	

* Invasive Margin ** Center of the Tumor.

**Table 2:** Multivariate analysis of the prognostic impact of CD3^+^ and CD8^+^ T-cell density at the invasive margin and center of the tumor for overall survival and progression free survival.

Prognostic Factor	Overall Survival		Progression Free Survival	
	Hazard Ratio (95% CI) *^p^*^-Value^	*p*-Value	Hazard Ratio (95% CI) *^p^*^-Value^	*p*-Value
IM CD3 density (cells/mm^2^)				
<827 vs. 827–2680	1.66 (1.08–2.55) *	0.017	1.47 (1.04–2.07) *	0.044
827–2680 vs. ≥2680	1.30 (0.77–2.19) ^n.s.^		1.13 (0.78–1.64) ^n.s.^	
CT CD3 density (cells/mm^2^)				
<123.5 vs. 123.5–821	2.15 (1.37–3.38) ***	0.004	1.79 (1.25–2.56) *	0.006
123.5–821 vs. ≥821	0.80 (0.50–1.28) ^n.s.^		0.88 (0.62–1.27) ^n.s.^	
IM CD8 density (cells/mm^2^)				
<245 vs. 245–1208.6	1.44 (0.88–2.36) ^n.s.^	0.29	1.30 (0.89–1.89) ^n.s.^	0.26
245–1208.6 vs. ≥1208.6	0.80 (0.33–0.51) ^n.s.^		0.80 (0.57–1.14) ^n.s.^	
CT CD8 density (cells/mm^2^)				
<36.6 vs. 36.6–409	1.36 (0.84–2.20) ^n.s.^	0.4	1.14 (0.77–1.68) ^n.s.^	0.5
36.6–409 vs. ≥409	0.82 (0.52–1.30) ^n.s.^		0.82 (0.58–1.15) ^n.s.^	

IM Invasive Margin, CT Center of the Tumor, * *p* ≤ 0.05, *** *p* ≤ 0.0001, over all *p*-value, n.s. = not significant.

## Data Availability

Data is contained within the article and Supplementary Materials and is available for bona fide researchers who request it from the authors.

## Supplementary Materials

The following supporting information can be downloaded at: https://www.mdpi.com/article/10.3390/cancers14174246/s1. Figure S1: Automated cell detection in manually annotated regions. Figure S2: Kaplan–Meier Estimates for progression-free survival in vulvar cancer. Figure S3: Kaplan–Meier Estimates for progression-free survival among patients with different immune phenotypes. Figure S4: Prognostic impact of the estimated CD4+ T-cell density for overall survival and progression-free survival. Table S1: Patient characteristics. Table S2: Association between the estimated density of CD4+ T-cells at the invasive margin/ in the center of the tumor and vulvar cancer phenotype. (±Standard deviation). Table S3: Multivariate analysis of the prognostic impact of the estimated CD4+ T-cell density at the invasive margin and center of the tumor for overall survival and progression free survival.
