# Supplementary material for: T-Cell Density at the Invasive Margin and Immune Phenotypes Predict Outcome in Vulvar Squamous Cell Cancer
Source: Cancers (Basel). 2022 Aug 31;14(17):4246. doi: 10.3390/cancers14174246 (PMC9454842; doi:10.3390/cancers14174246)
Supplement: Supplementary file 1 [file cancers-14-04246-s001.zip › cancers-1801155-supplementary.pdf]

Supplementary Appendix

Manuscript title: T-cell density at the invasive margin and immune phenotypes predict outcome in vulvar squamous cell cancer.

Section S1: Supplementary Figures S1 – S4 2

Figure S1: Automated cell detection in manually annotated regions. 2

Figure S2: Kaplan–Meier Estimates for progression-free survival in vulvar cancer. 3

Figure S3: Kaplan–Meier Estimates for progression-free survival among patients with different immune phenotypes. 4

Figure S4: Prognostic impact of the estimated CD4^{+} T-cell density for overall survival and progression-free survival 5

Section S2: Supplementary Tables S1-S3 6

Table S1: Patient characteristics 6

Table S2: Association between the estimated density of CD4^{+} T-cells at the invasive margin/ in the center of the tumor and vulvar cancer phenotype. (±Standard deviation). 7

Table S3: Multivariate analysis of the prognostic impact of the estimated CD4^{+} T-cell density at the invasive margin and center of the tumor for overall survival and progression free survival. 8

Section S1: Supplementary Figures S1 - S4

Figure S1: Automated cell detection in manually annotated regions.
A trained pathologist has annotated the invasive margin (IM, red) and the center of tumor (CT, blue) so that a machine learning algorithm could be used for cell segmentation in both an "immune desert" (a) as well as "immune inflamed" phenotype (b) to assess the number of positive stained T-cells. The number of positive cells and the size of the annotate region were used to calculate the density of stained cells (number of cells per square mm) for every patient.

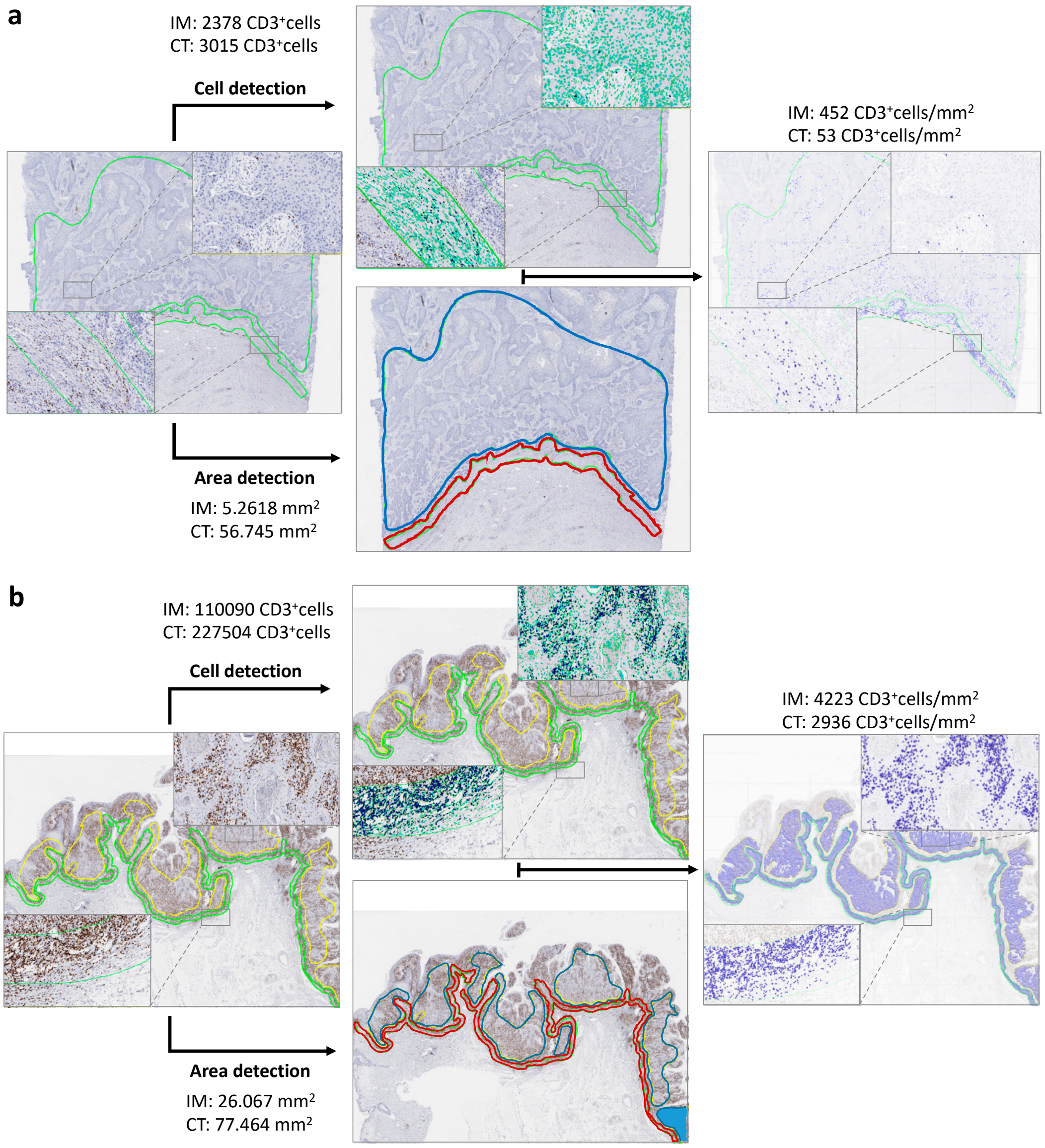

Figure S2: Kaplan–Meier Estimates for progression-free survival in vulvar cancer.

Prognostic impact of the $CD3^+$ and $CD8^+$ T-cell density at the invasive margin (a, b) as well as in the center of the tumor (c, d).

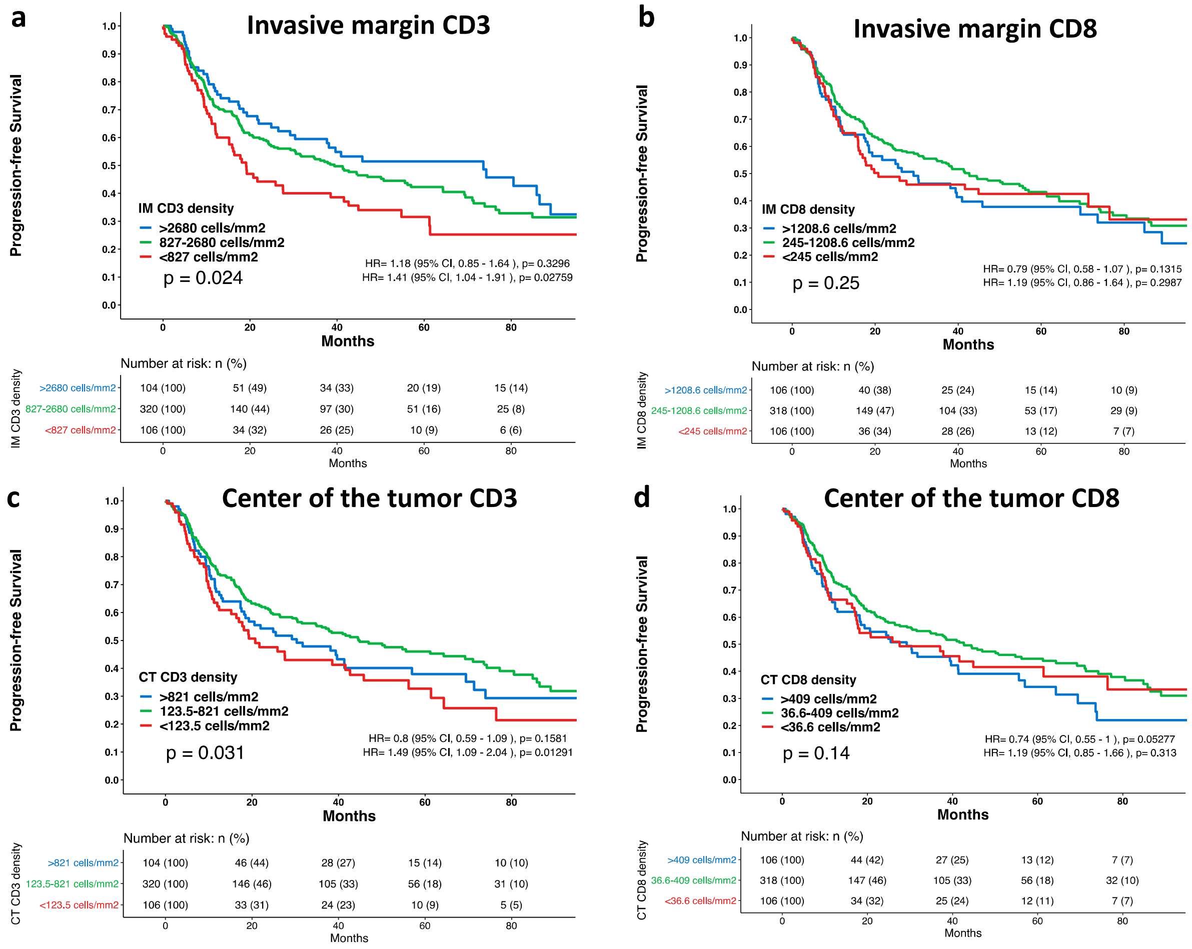

Figure S3: Kaplan–Meier Estimates for progression-free survival among patients with different immune phenotypes.

Prognostic impact of the clustered (a) and histomorphological identified (b) immune phenotypes.

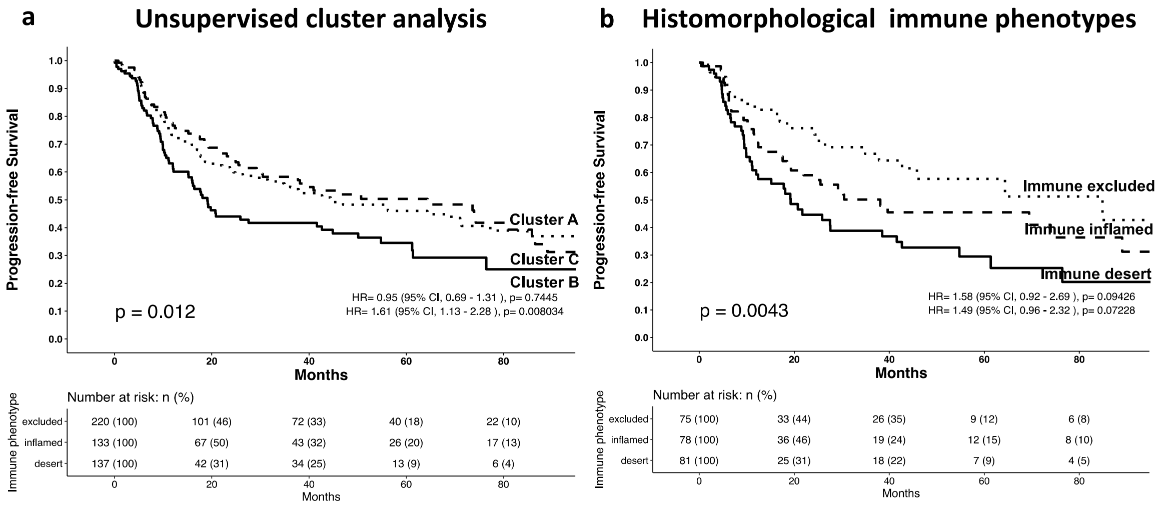

Figure S4: Prognostic impact of the estimated CD4+ T-cell density for overall survival and progression-free survival

Kaplan-Meier estimates for the prognostic impact of the estimated CD4+ T-cell density at the invasive margin and the center of the tumor for overall survival (a,b) and progression free survival (c,d).

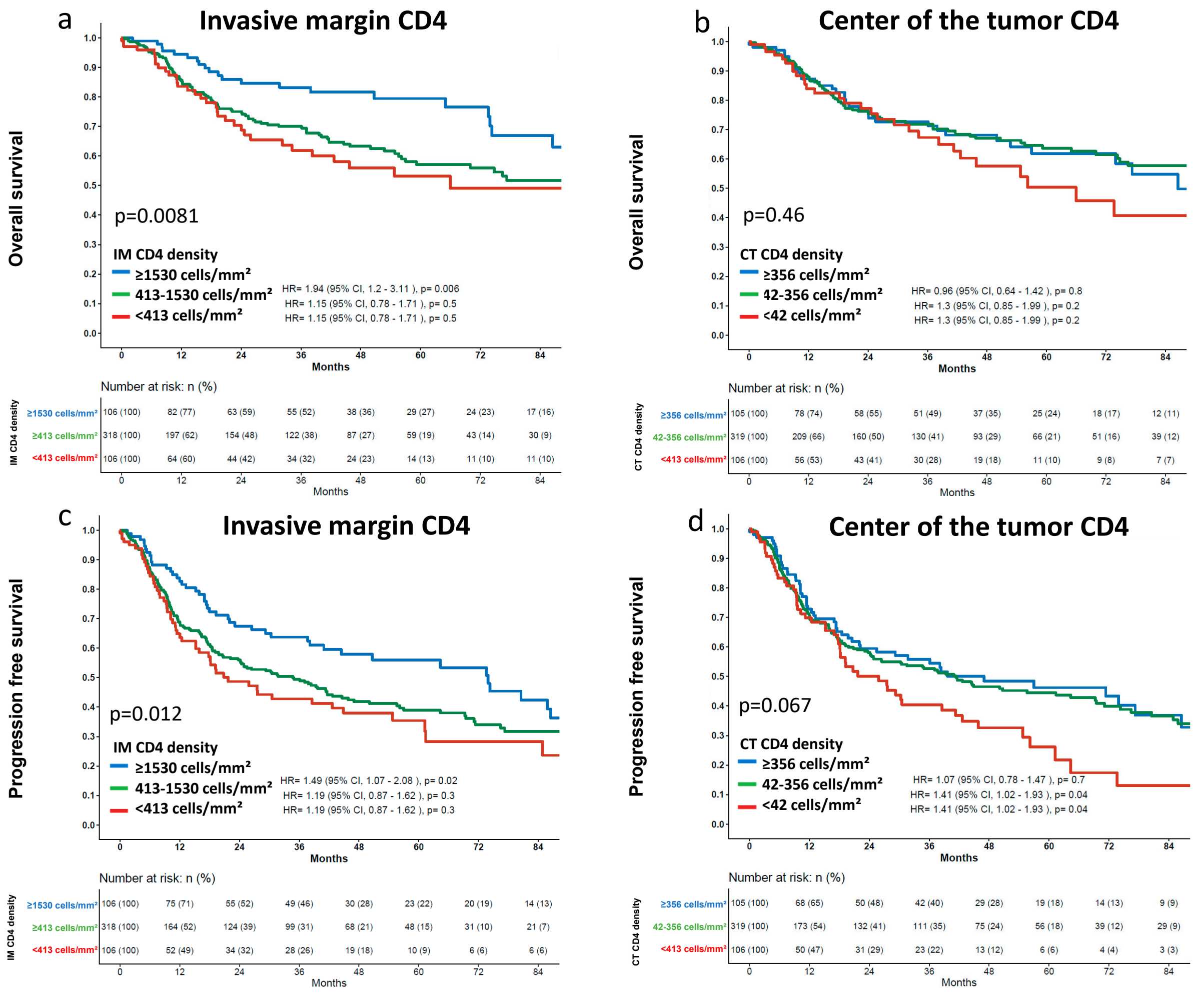

Section S2: Supplementary Tables S1 - S3

Table S1: Patient characteristics

Patients characteristics	No. of patients (%)		
	Total study cohort on TMA (n=637)	Overall death among categories	Overall progression among categories
Follow-up - no.	637 (100.0%)	181 (28.4%)	303 (47.6%)
Median - months (95% confidence interval)	22 (19-26)	-	-
Age (median) - years	70	-	-
pT stage - no. %			
pT1	206 (32.3%)	33 (18.2%)	72 (23.8%)
pT2	347 (54.5%)	117 (64.6%)	181 (59.7%)
pT3-4	74 (11.6%)	27 (14.9%)	42 (13.9%)
missing data	10 (1.6%)	4 (2.2%)	8 (2.6%)
pN stage - no. %			
pN0	339 (53.2%)	62 (34.3%)	118 (38.9%)
pN+	219 (34.4%)	91 (50.3%)	139 (45.9%)
missing data	79 (12.4%)	28 (15.5%)	46 (15.2%)
Grade - no. %			
1	74 (11.6%)	21 (11.6%)	36 (11.9%)
2	383 (60.1%)	94 (51.9%)	173 (57.1%)
3	169 (26.5%)	65 (35.9%)	89 (29.4%)
missing data	11 (1.7%)	1 (0.5%)	5 (1.7%)
HPV - no. %			
HPV0	299 (46.9%)	97 (53.6%)	156 (51.5%)
HPV+	306 (48%)	72 (39.8%)	130 (42.9%)
missing data	32 (5%)	12 (6.6%)	17 (5.6%)

Table S2: Association between the estimated density of CD4+ T-cells at the invasive margin/ in the center of the tumor and vulvar cancer phenotype. (±Standard deviation).

Clinical parameter	n	Density of CD4+ cells (IM*) [cells/mm²]	p-value	Density of CD4+ cells (CT**) [cells/mm²]	p-value
pT1	174	1209 (±795)	<0.0001	285 (±283)	0.0125
pT2	286	957 (±656)		213 (±266)	
pT3-4	64	778 (±585)		198 (±267)	
pN-	285	1081 (±708)	0.0235	238 (±260)	0.7816
pN+	180	928 (±698)		231 (±284)	
Grade 1	60	1208 (±776)	0.0890	251 (±333)	0.2048
Grade 2	317	1000 (±699)		218 (±233)	
Grade 3	145	983 (±708)		265 (±323)	
HPV-	244	1014 (±698)	0.7978	238 (±303)	0.8796
HPV+	259	1030 (±717)		234 (±246)	

*Invasive Margin; **Center of the Tumor

Table S3: Multivariate analysis of the prognostic impact of the estimated CD4+ T-cell density at the invasive margin and center of the tumor for overall survival and progression free survival.

**Prognostic Factor**	**Overall Survival**		**Progression free survival**	
	Hazard Ratio (95% CI)	p-value	Hazard Ratio (95% CI)	p-value
IM CD4 density (cells/mm^2)				
<413 vs. 413-1530	1.30 (0.83 - 2.04)$^{n.s.}$	0.042	1.21 (0.84 - 1.73)$^{n.s.}$	0.011
413-1530 vs. ≥1530	1.64 (0.98 - 2.75)$^{n.s.}$		1.63 (1.11 - 2.39)*	
CT CD4 density (cells/mm^2)				
<42 vs. 42-356	1.54 (0.96 - 2.46)$^{n.s.}$	0.142	1.50 (1.04 - 2.16)*	0.087
42-356 vs. ≥356	0.73 (0.46 - 1.16)$^{n.s.}$		0.95 (0.66 - 1.36)$^{n.s.}$	

*p≤0.05, [ ]:over all p-value, n.s.=not significant
